# Cellular reprogramming for understanding and treating human disease

**DOI:** 10.3389/fcell.2014.00067

**Published:** 2014-11-12

**Authors:** Riya R. Kanherkar, Naina Bhatia-Dey, Evgeny Makarev, Antonei B. Csoka

**Affiliations:** ^1^Epigenetics Laboratory, Department of Anatomy, Howard UniversityWashington, DC, USA; ^2^InSilico Medicine, Emerging Technology Center, Johns Hopkins University EasternBaltimore, MD, USA

**Keywords:** reprogramming, stem cells, aging, disease, epigenetics

## Abstract

In the last two decades we have witnessed a paradigm shift in our understanding of cells so radical that it has rewritten the rules of biology. The study of cellular reprogramming has gone from little more than a hypothesis, to applied bioengineering, with the creation of a variety of important cell types. By way of metaphor, we can compare the discovery of reprogramming with the archeological discovery of the Rosetta stone. This stone slab made possible the initial decipherment of Egyptian hieroglyphics because it allowed us to see this language in a way that was previously impossible. We propose that cellular reprogramming will have an equally profound impact on understanding and curing human disease, because it allows us to perceive and study molecular biological processes such as differentiation, epigenetics, and chromatin in ways that were likewise previously impossible. Stem cells could be called “cellular Rosetta stones” because they allow also us to perceive the connections between development, disease, cancer, aging, and regeneration in novel ways. Here we present a comprehensive historical review of stem cells and cellular reprogramming, and illustrate the developing synergy between many previously unconnected fields. We show how stem cells can be used to create *in vitro* models of human disease and provide examples of how reprogramming is being used to study and treat such diverse diseases as cancer, aging, and accelerated aging syndromes, infectious diseases such as AIDS, and epigenetic diseases such as polycystic ovary syndrome. While the technology of reprogramming is being developed and refined there have also been significant ongoing developments in other complementary technologies such as gene editing, progenitor cell production, and tissue engineering. These technologies are the foundations of what is becoming a fully-functional field of regenerative medicine and are converging to a point that will allow us to treat almost any disease.

## Introduction

### Paradigm shifts and seeing things in new ways

Stem cell biology is sometimes thought of as a field that offers promises that can't be kept; for example the potential to one day treat heart attacks or repair spinal cord injuries. But focusing on regenerative medicine's so-far small impact on patient care, misses a shift in our understanding of cells so radical that it has rewritten the rules of biology in less than two decades.

**Figure 1:**
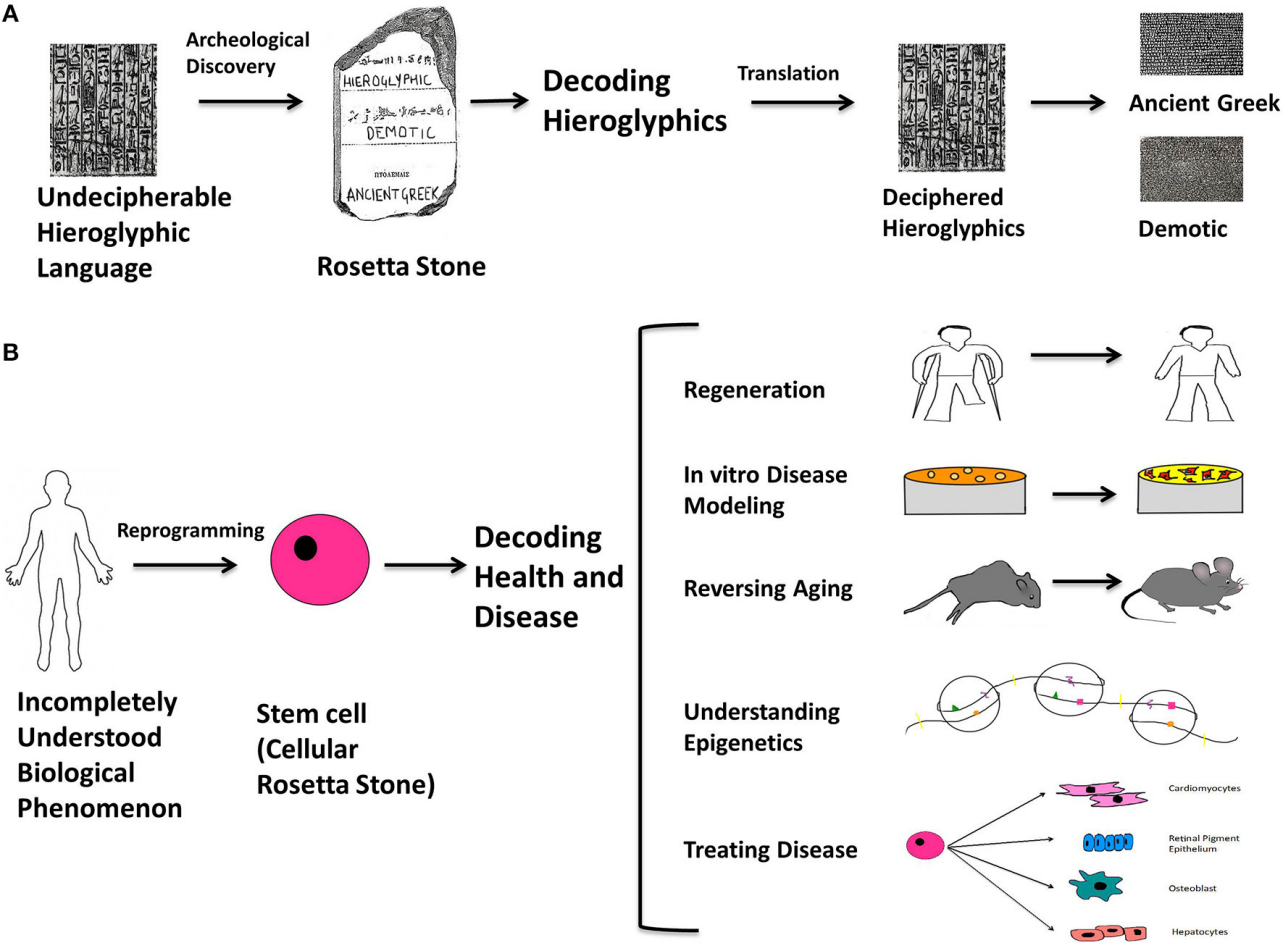
**The Rosetta stone analogy**. The Rosetta stone is an archeological slab with the same text in three different languages; Hieroglyphics, Demotic, and Ancient Greek, and its discovery proved to be a turning point in understanding the Hieroglyphic language. By direct comparison of the three languages, it was possible to decipher previously unintelligible Hieroglyphics from the other two known languages. An analogy can be made with respect to stem cells: they can be thought of as “cellular Rosetta stones” because they are key to understanding the multi-faceted mysteries underlying human health and disease. Stem cells can be conceptualized as “cellular Rosetta stones” because they are enabling us to create and compare cells with diseases or cellular phenomena that are poorly understood (non-deciphered Hieroglyphics), with isogenic cells that are completely normal (well-understood, Ancient Greek or Demotic). This is the contribution of stem cells toward modeling and treating diseases and generating functional cell types. In addition, they are becoming quintessential for studying epigenetics, aging, cancer and regeneration. It won't be long before they emerge as the ultimate practical tool and make their mark on the conceptualization of biological science in terms of understanding molecular and cellular events and treating debilitating diseases. By using reprogramming as the means to make cellular Rosetta stones, it will be possible to form a universal understanding of currently unknown biological phenomena and develop an accurate philosophy for cellular processes, disease and therapy. **(A)** Illustrates the rationale behind the way the Rosetta stone was used to decode hieroglyphics. **(B)** Illustrates the analogously unexplained biological phenomena to which stem cells can be made with various characteristics on an isogenic background and used to understand them; for example (1) limb-regeneration (2) disease modeling at the cellular level (3) treating organismal aging (4) understanding epigenetic mechanisms underlying diseases (5) generating lineage specific cell types to treat degenerative and chronic diseases, or acute injuries.

How much have things changed? By way of analogy, the Rosetta stone was a stone slab found in 1799 that bore parallel inscriptions in Greek, Demotic characters, and Egyptian hieroglyphics that made possible an unprecedented decipherment of the latter. We propose that the discovery of the mechanisms for producing and modulating stem cells, in particular induced pluripotent stem cells, will have an equally profound impact in understanding human health and disease (Figure 1). Stem cells can be conceptualized as “cellular Rosetta stones” because they are enabling us to create and compare cells with diseases or cellular phenomena that are poorly understood (non-deciphered Hieroglyphics), with isogenic cells that are completely normal (well-understood, Ancient Greek or Demotic). Because of this, reprogrammed stem cells will be the quintessential tool for studying epigenetics, aging, cancer, and regeneration. This new possibility will open up opportunities to study cells that go awry in disease and to one day use patients' own cells to heal them.

Stem cells are emerging as the ultimate tool for understanding biological processes at the molecular and cellular level, as well as treating debilitating diseases. By using reprogramming as the means to make “cellular Rosetta stones,” it will be possible to form an accurate understanding of currently unknown biological phenomena, and perhaps eventually develop a universal synthesis of cell biology (Figure 1).

In this review we present a comprehensive summary of the history of cellular reprogramming from its initial conception to the present day. We also describe how these techniques are being used to better understand basic biology and human disease, and lay the foundations for a fully functional discipline of regenerative medicine.

Therefore, to begin, what exactly are stem cells?

### Stem cells

Stem cells are undifferentiated cells that possess two unique characteristics:

1. The ability to self-renew through numerous cycles of cell division while maintaining an undifferentiated state, and
2. Pluripotent potential with the ability to generate progenitors of multiple lineages (International Stem Cell et al., 2007; Mitalipov and Wolf, 2009). These qualities confer unique regenerative abilities upon stem cells, and make them a desirable commodity in the endeavor to replenish, regenerate and repair human tissues (Gurtner et al., 2007; Wu et al., 2007). There are essentially two types of stem cells in mammals: somatic stem cells (SSCs, also sometimes called progenitor cells), and embryonic stem cells (ESCs) (Niwa et al., 2000).

SSCs are found in various adult tissues, such as bone marrow, adipose tissue and blood (including umbilical cord blood) (Jiang et al., 2002; Terai et al., 2006; Gimble et al., 2007; Luna-Zurita and Bruneau, 2013). They are partially differentiated cells found throughout the body that possess the capacity to divide in order to replenish damaged tissue. They are able to differentiate into more than one cell type, but unlike ESCs, they are restricted to a specific cellular lineage. The advantage of SSCs is that their production doesn't require the destruction of embryos, or reprogramming (see below) (Gardner, 2002).

ESCs are produced from eggs derived from the inner cell mass of fertilized embryos. Contrasted with SSCs, ESCs are pluripotent, and can differentiate into *all* of the three primary germ layers (ectoderm, endoderm, and mesoderm) and their derivatives. ESCs are characterized by long-term self-renewal, and can be grown in cell culture as an undifferentiated, pluripotent population. Regulation of pluripotency networks is important for maintaining the undifferentiated state of such cells in culture, or during differentiation to obtain desired cell types. The transcription factor (TF), Oct 3/4 is the master regulator of pluripotency, and its precise levels during development are responsible for the differentiation of ESCs into specific lineages, whereas repression of Oct 3/4 results in loss of pluripotency and formation of trophoectoderm (Niwa et al., 2000).

ESCs can be directed to differentiate into a particular cell type through alteration of culture conditions and/or the supplementation of differentiation signals. Understanding the differentiation process has provided insights into de-differentiation and trans-differentiation strategies as well. Dedifferentiation is the formation of pluripotent or multipotent stem cells from terminally differentiated somatic cells, i.e., reverting to a state of increased developmental plasticity, and becoming ready to accept a new identity (Halley-Stott et al., 2013). Transdifferentiation is the process in which a particular somatic cell is switched from one lineage-specific identity to a completely different identity (Graf, 2011; Vierbuchen and Wernig, 2012); in other words, the direct conversion of one type of somatic cell into another type, bypassing the intermediate step of dedifferentiation.

The discovery of ESCs (Evans and Kaufman, 1981; Martin, 1981) eventually prompted the search for discovering artificial dedifferentiation techniques to confer the properties of ESCs onto somatic cells by altering epigenomic activity, such that the derived cells are pluripotent and capable of giving rise to embryonic-like stem cells. These techniques are collectively referred to as cellular reprogramming. But before we describe these various techniques, we will provide some background on the history of how we arrived at today's reprogramming technology.

## History and development of cellular reprogramming

In 1909, Ethel Browne Harvey, who was known for her work on sea urchins, was the first to show that cell transplants could induce a secondary axis of polarity in the host. Harvey's experiments were the basis for the discovery of Spemann's organizer (Lenhoff, 1991). In 1928, Hans Spemann and Hilde Mangold, in a quest to discover the factors responsible for embryonic determination and cell differentiation, performed classical embryology experiments with salamanders and demonstrated cell-to-cell induction, in which a group of cells or organizing centers signal differentiation in neighboring cells and hence regulate their fate in the embryo (De Robertis, 2006). The cells responsible for this kind of phenomenon came to be known as the Spemann organizer, which over subsequent decades led to many experiments in molecular embryology aimed at finding inducing factors responsible for early embryonic determination and cell fate (Grunz, 2001). Further, Spemann had proposed an experiment to determine whether differentiated cells could be restored to an embryonic state, or if the cells continued to remain specialized (Subramanyam, 2013). Spemann reasoned that if a nucleus from a differentiated cell implanted in a previously enucleated egg developed into a normal embryo, this would prove that the transplanted nucleus retained a genome fully capable of directing all types of differentiation. In other words, a differentiated nucleus could still be totipotent.

### Somatic cell nuclear transfer

In 1938, Spemann published an account of his experiments with a prototypical nuclear transfer technique (Spemann, 1938). Using a piece of hair wrapped around a newly-fertilized salamander egg, he separated the egg's nucleus on one side, with the cytoplasm on the other. After the nucleated side divided four times, creating a 16-cell embryo, he removed the hair and allowed a nucleus to slide back into the separated cytoplasm. Cell division now began on this side as well, and by putting the hair loop back again tightly, he broke the two embryos apart. The result was a twin set of salamanders.

This important work showed that the nucleus remained totipotent after undergoing four divisions, but Spemann wondered whether nuclei from much older embryos, or even adult animals, had similar potential. He wrote that transplanting an older nucleus into an egg would be a “fantastical experiment.” What he was proposing is what eventually became known as somatic cell nuclear transfer (SCNT). However, for the next 14 years, scientists struggled with making it work.

But in 1952 Briggs and King performed the first successful SCNT experiment showing the reversal of cellular identity. They showed that the transfer of a frog nucleus from a blastula cell to an enucleated egg gave rise to a cleaved blastula (Briggs and King, 1952). This was followed by the pioneering experiments of John Gurdon in 1962, involving transfer of nuclei from terminally differentiated frog intestinal epithelium cells into enucleated eggs to produce normal tadpoles, which proved that the nucleus contains all the genetic information required to give rise to all the differentiated cells in the organism (Gurdon, 1962). After incremental improvements in SCNT techniques by various laboratories over the intervening three decades, the cloning of “Dolly” in 1996 was a major breakthrough, and the first ever successful mammalian cloning experiment, which involved transplanting quiescent nuclei from cultured adult sheep mammary gland cells into enucleated sheep eggs (Campbell et al., 1996). The creation of SCNT-derived human ESCs required further improvements in technology and almost another two decades, but in 2013, after a long and challenging pursuit, Mitalipov and colleagues finally succeeded in creating SCNT-derived human embryonic stem cell (hESC) lines (Tachibana et al., 2013).

SCNT has, over the decades, evolved as a technique, which can be used for reproductive cloning as well as to produce lines of hESCs. The attraction of using an egg for nuclear reprogramming is that, in nature, it has near 100% efficiency in reprogramming sperm nuclei (Gurdon and Melton, 2008; Teperek and Miyamoto, 2013). The egg uses molecular chaperones and enzymes to erase the epigenetic signatures during synkaryon formation and cell division, resulting in extremely efficient reprogramming (Kikyo and Wolffe, 2000). Variations in the technique used to produce offspring through SCNT such as improvement of processes like oocyte maturation, enucleation, donor nucleus transfer, activation and culture, along with prior epigenetic modification of the donor nucleus, can improve the efficiency of the process (Campbell et al., 2007).

### Induced pluripotency

Once SCNT in mammals was achieved, researchers wondered if the same result could be achieved without using eggs. Thanks to the pioneering experiments of Yamanaka, it is now possible to create close equivalents of ESCs without using embryos by engineering their creation *in vitro*. In 2006 and 2007, Yamanaka and colleagues identified conditions that allowed somatic cells to be genetically reprogrammed into induced pluripotent stem cells (iPSCs) that are almost as potent as ESCs. This was a significant breakthrough and was achieved by transfection of mouse and human fibroblasts with the transcription factors (TFs) Oct4, Sox2, Klf4, and c-Myc, (OSKM) using retroviral vectors to induce a “forced” expression of specific genes that results in iPSC formation (Takahashi and Yamanaka, 2006; Okita et al., 2007). Subsequent manipulation using different reprogramming factors such as Nanog rendered the reprogramming process more efficient (Wernig et al., 2007). There are now many different such pathways of inducing pluripotency, because different combinations of reprogramming factors can all achieve complete reprogramming (Takahashi, 2012). In addition to such reprogramming factors, introduction of improvements such as p53 knockdown and telomerase overexpression, along with small molecules that exert epigenetic effects (see Section Small Molecules below) are now being used routinely (Batista, 2014; Campos-Sanchez and Cobaleda, 2014). Besides using different combinations of TFs, iPSCs have also been induced using methods other than retroviral vectors, such as DNA and RNA (integrating plasmids, episomal plasmids and transposons), cell penetrating peptides, and small molecules (see Small Molecules below) (Li et al., 2014).

At present, a primary concern is the ability to achieve pluripotency in iPSCs while maintaining the functionality of ESC's—irrespective of the type of somatic cells used at the outset. For example, it is possible that less differentiated cells of the hematopoietic lineage reprogram more efficiently than differentiated cells, suggesting that the use of SSCs/progenitor cells as starting material could maximize the efficiency of reprogramming (Eminli et al., 2009). This may be because less differentiated progenitors possess less condensed chromatin in specific regions, which is more accessible to reprogramming factors. Also progenitors in general may be more susceptible to the disruption of their transcriptional networks, or possess a transcriptome that has higher resemblance to the transcriptome of ESCs (Papp and Plath, 2011).

A very recent refinement to induced pluripotency has been achieved which allows the *in vitro* reprogramming of cells back to a ground state of pluripotency that is even closer to ESCs. Novel human naïve stem cells have been derived by adding a unique combination of cytokines and small molecule inhibitors to the standard reprogramming cocktail (Gafni et al., 2013). These ground state stem cells closely resemble mouse iPSCs and ESCs (relatively more pluripotent than human ESCs or iPSCs), exhibiting hallmarks of naïve pluripotency that include driving Oct 4 transcription by its distal enhancer, retaining a pre-inactivation X chromosome state and a global reduction in DNA methylation and an H3K27me3 repressive chromatin mark deposition on developmental regulatory gene promoters (Gafni et al., 2013). The epigenetic changes induced by naïve human stem cell medium (NSHM) conditions indicated that naïve conditions are likely to resolve previously described technical phenotypes of epigenetic memory, lineage differentiation biases and aberrant reprogramming in human iPSCs and ESCs, thus providing a stable source for treating and modeling diseases (Gafni et al., 2013).

Also, recent studies in mice have shown that the *in vivo* transitory induction of OSKM generated teratomas, indicating that *in situ* reprogramming can give rise to iPSCs from a variety of cell types, and these *in vivo* iPSCs more closely resembled ESCs than *in vitro* generated iPSCs, with a more primitive and plastic state (Abad et al., 2013) (**Figure 3**). These *in vivo* iPSCs can further generate desired cell types in the presence of appropriate signals for re-differentiation (shown in **Figure 3**).

The most attractive application of induced pluripotency using the newest techniques is of course the production of patient-specific iPSCs for replacement of damaged, aged, or non-functional tissue, which will be discussed in later sections. While both ESCs and iPSCs are desirable for reasons of potency, the availability of ESCs is hindered by practical (egg availability) and ethical concerns, while iPSC production still remains relatively inefficient.

### Cell fusion

Another possible alternative to SCNT is cell fusion, in which two cells fuse together in the presence of agents like Sendai virus, polyethylene glycol (PEG), chimeric hemagglutinins, or an electric pulse to give rise to homokaryons from the same type of cells or heterokaryons from different types of cell (Soza-Ried and Fisher, 2012). In the case of heterokaryon formation, the dominant cell is the larger and actively dividing partner that imposes its own pattern of gene expression on the other. But cells fused in this way do not usually proliferate well, limiting their therapeutic value (Gurdon and Melton, 2008).

### Cell-extract treatment

In recent years, cell extracts (usually extracted from embryonic-like stem cells) have been used to derive pluripotent cells that can give rise to diverse cell lineages following differentiation (Patel and Yang, 2010). The use of extracts derived from pluripotent cells such as ESCs can also trigger the formation of ESC-like colonies with the upregulation of pluripotency genes and the downregulation of somatic genes such as Lamin A (*LMNA*) (Alberio et al., 2006). Notably, remodeling of the nuclear lamina has been used as a marker for reprogramming events in experiments involving the incubation of *Xenopus laevis* somatic cells in egg-extract in which reprogramming events could be tracked by the resulting nuclear configuration which acted as an indicator of increased plasticity (Alberio et al., 2006). *LMNA* is also a marker of differentiation of human ESC's into somatic cells (Constantinescu et al., 2006).

### Small molecules

Another recent addition to the different types of reprogramming techniques is the use of small molecules, with or without a combination of TFs. It is thought that small molecules might lead to a better clinical approach because they involve fewer genetic manipulations than TF-based reprogramming (Pandian et al., 2014). A recent breakthrough study showed that a combination of just seven small-molecules was enough to chemically reprogram mouse embryonic fibroblasts (MEFs) such that Oct4 was dispensable for reprogramming (Hou et al., 2013). Such chemically induced pluripotent stem cells (CiPSCs) were similar to ESCs in terms of gene expression profile and epigenetic state, and did not require any exogenous expression of master pluripotency genes at all (Hou et al., 2013).

### Transdifferentiation/direct reprogramming

Transdifferentiation techniques might make it possible to produce patient-specific cells in a faster and more efficient manner by avoiding the intermediate stage of iPSC formation, and thus preventing the risk of tumorigenesis and immunogenicity associated with iPSCs (Ma et al., 2013) (**Figure 3**). For example, transdifferentiation studies *in vivo* have already demonstrated the conversion of adult murine pancreatic exocrine cells to β-cell-like populations using the TFs Ngn3, Pdx1, and Mafa (Zhou et al., 2008).

iPSC TF-based transdifferentiation is a new method of transdifferentiation that uses the transient overexpression of iPSC related TFs and cell-type-specific signals (growth factors, cytokines, and small molecules) to reprogram somatic cells to diverse lineage-specific cells or multipotent progenitors without transitioning through the iPSC stage (Ma et al., 2013). An example of such inter-lineage transdifferentiation is the generation of expandable neural progenitors from fibroblasts by the transient induction of OSKM along with appropriate signaling inputs (Kim et al., 2011). Another example is the conversion of human dermal fibroblasts to multipotent hematopoetic progenitors of myeloid, erythroid and megakaryocytic lineages via induction of OCT4 and its specific binding to regulatory regions of hematopoetic-specific genes along with cytokine supplementation (Szabo et al., 2010).

### Regeneration

Finally, the process of regeneration involves local dedifferentiation of somatic cells into a blastema composed of SSCs, which are multipotent and capable of proliferating, and re-differentiating into different lineages, and which can repopulate the damaged or degenerated tissue with functional cells. In human adults somatic cells cannot normally dedifferentiate and the process of wound healing involves scar tissue formation caused by the deposition of collagen, which is the most abundant part of the extracellular matrix secreted during wound healing. Thus, as compared to lower vertebrates like salamanders that are capable of complete regeneration with very little or no scar tissue, human adults have limited regenerative capacity. Regenerative capacity in humans seems to have diminished during the course of evolution compared to our lower vertebrate cousins as a mechanism to fight cancer.

However, regeneration in humans is not completely non-existent. The developing fetus is capable of scarless wound healing and this capacity is lost in gestation. For example, in the case of cardiac regeneration in neonates, the same extracellular matrix that contributes to scar formation might be involved in signaling an increase in proliferative capacity (Porrello and Olson, 2014). Thus, the much-coveted mammalian fetal regeneration capacity including wound healing without scar-tissue formation is a result of the extracellular matrix modulators, growth factors as well as cytokines (Colwell et al., 2005).

In spite of having wide-ranging information about the regenerative capacity of invertebrates, absolute knowledge of the limits of mammalian regenerative capacity is obscure because our understanding of mammalian stem cell biology usually comes from isolated stem cells, rather than from actually studying them *in vivo*. Given the fact that cells are dynamic entities and their properties change according to the environment they are in, the actual information on the number and functionality of stem cells *in vivo* is not accurately known (Sanchez Alvarado and Yamanaka, 2014).

Apart from harvesting the intrinsic regenerative potential of stem cells, mammalian regeneration can be induced through the administration of TFs known to locally dedifferentiate cells (transient) followed by re-differentiation. In a landmark study, it was shown that the upregulation of a single transcription factor, FOXN1, resulted in regeneration of the thymus in aged mice to an extent that it restored thymopoesis, a ground breaking example of *in vivo* mammalian regeneration (Bredenkamp et al., 2014). Such recent efforts in regeneration research have helped to advance our knowledge to the point where restoration of regenerative potential in mammals is perhaps within reach.

All of the above cellular reprogramming methods remodel chromatin and cause epigenetic changes in the target cells. But before we describe the application of the above methods to understanding and treating disease, we will first provide an explanation of what exactly is meant by “epigenetics,” with a brief summary of this rapidly developing field.

## Epigenetics

### Overview of epigenetics

Epigenetics is the study of heritable changes in gene activity exclusive of direct modification of the DNA sequence, and includes DNA methylation and post-translational histone modification (Berger et al., 2009). The epigenome is a collection of the DNA methylation states and covalent modifications of histone proteins along the genome, and it differs in each cell type. Epigenetic mechanisms play an important role in the control of gene expression by organizing the nuclear architecture of chromosomes, restricting or facilitating TF access to DNA, and preserving a memory of past transcriptional activities (Rivera and Ren, 2013).

Epigenetic theory explains how the genome and environment work in tandem, involving mechanisms that affect DNA by regulating gene expression (Weinhold, 2006), and this interaction can operate across the entire human lifespan (Kanherkar et al., 2014).

**Figure 2:**
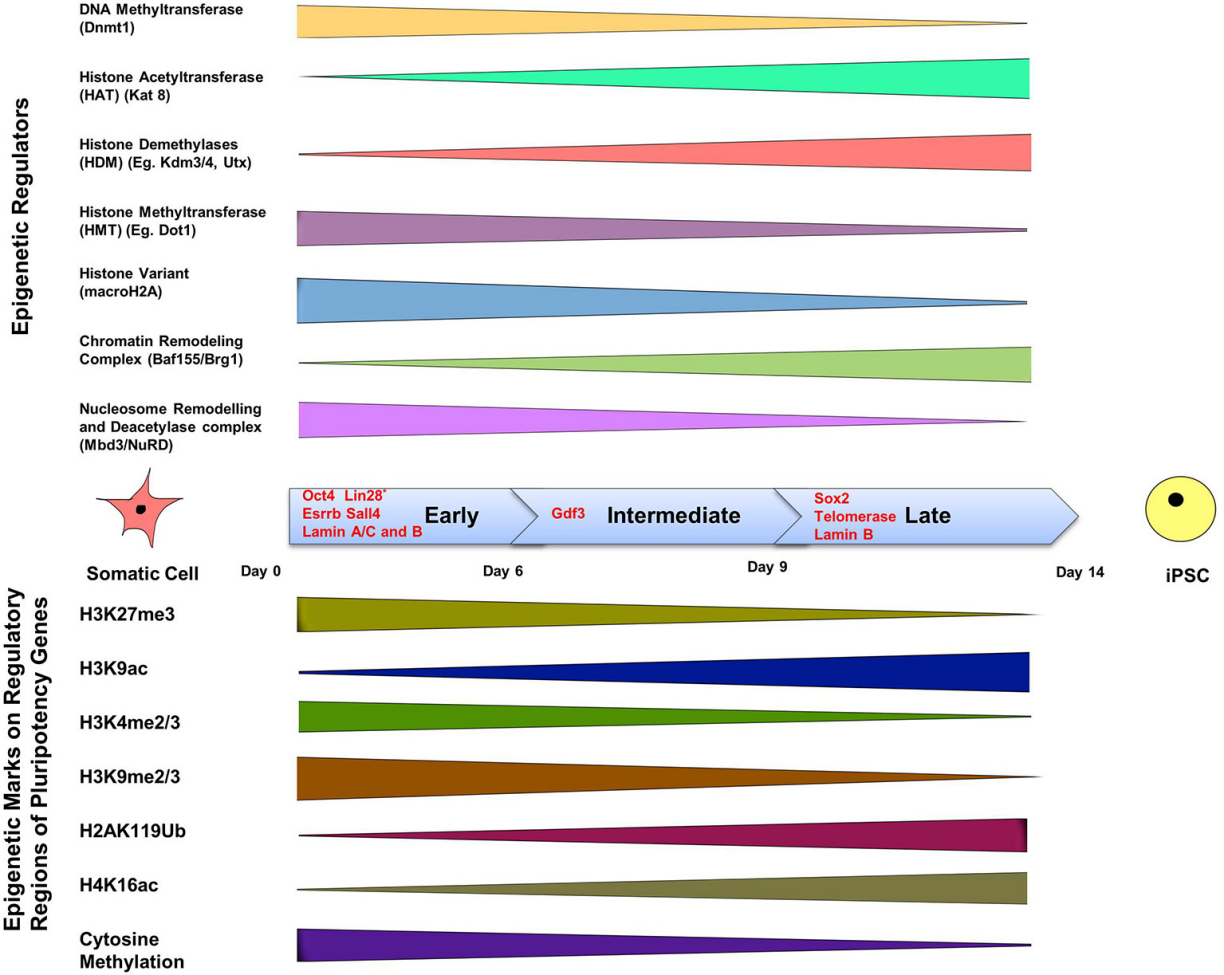
**The epigenetics of induced pluripotency**. The process of induced pluripotency using OSKM involves turning on pluripotency genes and turning off genes responsible for the maintenance of a differentiated somatic cell state. The timeline of formation of an iPSC can be divided into three stages; early, intermediate, and late, and involves the presence of different transcription factors during each of these phases. Transcription factors Oct4, Esrrb, and Sall4, are expressed during the early stage. Lin 28, suggested to be expressed in the early phase, is a controversial marker of early stages of reprogramming. Gdf3 is expressed during the intermediate stage, and Sox2 during the late stage. Lamins A/C and B are expressed in somatic cells but not in iPSCs, whereas telomerase expression is upregulated in iPSCs, but not in somatic cells. The color bars tapering toward either side of the timeline indicate a decline in expression or activity of the epigenetic regulator or epigenetic mark. The top panel shows the range of activity of some well-defined epigenetic regulators of chromatin in a somatic cell during the three phases. Methyl group-adding enzymes are shown on the very top, next is histone acetyltranferase that adds acetyl groups, followed by demethylases that remove methylation marks; the presence of histone variant: macroH2A, and finally chromatin remodeling complexes are depicted in the last two rows. The bottom panel includes the list of known epigenetic tags present on regulatory regions of pluripotency genes and their presence during the early, late and intermediate phases of induction in a somatic cell to pluripotency using reprogramming factors. DNA methylation on pluripotency genes decreases in the course of reprogramming because of a decline in the activity of Dnmt (DNA Methyltransferase), which is responsible for methylation. Histone acetylation increases during reprogramming due to the increased activity of histone acetyltransferase. Insufficient histone acetylation and hypermethylated DNA are the “epigenetic barriers,” which need to be overcome during reprogramming. Reprogramming leads to acquisition of active histone marks (e.g., H3K9ac) and loss of repressive histone marks (H3K4me2) on pluripotency genes, which facilitates the opening up of a compact chromatin structure and thereby allowing exposure of pluripotency gene promoters and binding of pluripotency factors like Oct4. (Buganim et al., 2012; Hansson et al., 2012; Loh and Lim, 2012; Apostolou and Hochedlinger, 2013; Liang and Zhang, 2013; Luna-Zurita and Bruneau, 2013; Papp and Plath, 2013).

Epigenetic modifications are of course crucially important for driving a cell toward an appropriate function during differentiation. The complexity of signaling during differentiation causes a cell's DNA to acquire specific epigenetic marks that restrict the expression of specific genes, and this inactivation survives cell division. DNA methylation is a signaling tool that occurs naturally on cytosine bases at CpG island promoter sequences and inactivates genes (Phillips, 2008). This type of epigenetic modification is associated with regulation of gene transcription, X-chromosome inactivation, and regulation of cellular development and differentiation (Bird, 2007). Another method of gene regulation is through the remodeling of chromatin. Remodeling occurs by post-translational modification of the amino acids that make up histone proteins via acetylation, methylation, phosphorylation and ubiquitination (Lunyak and Rosenfeld, 2008). Such epigenetic signatures are maintained by histone modifying enzymes such as histone acetyltransferases and histone methyltransferases (known as the “writers”) and histone demethylases and histone deacetylases (known as the “erasers”) which act as co-activators or co-repressors of OSKM respectively at different stages of reprogramming, and thereby influence iPSC formation (Apostolou and Hochedlinger, 2013) (Figure 2).

### Epigenetics during cellular reprogramming

Cellular reprogramming affects epigenetics at two different levels: firstly, through histone modification at the level of chromatin, and secondly through regulation of epigenetic marks on pluripotency genes and lineage-specifying genes that promote differentiation of various progenitors cells (Festuccia et al., 2013; Van Oevelen et al., 2013; Watanabe et al., 2013) (Figure 2). A major mechanism affecting epigenetic plasticity is genome-wide methylation and its regulation (Watanabe et al., 2013); nevertheless, it has to be accompanied by other epigenetic modulators that affect the state of pluripotency, totipotency, and their erasure (Loh and Lim, 2012). These modulators include histone acetylases and deacetylases such as Brg1 and Baf155 (Figure 2). While initial efforts to improve the reprogramming efficiency targeted the roles of transcriptional regulators, current studies suggest the involvement of signaling pathways in this process, and that the upregulation and downregulation of major signaling pathways can help in improving pluripotency reprogramming, lineage reprogramming and/or cell differentiation (Fritz et al., 2014).

**Figure 3:**
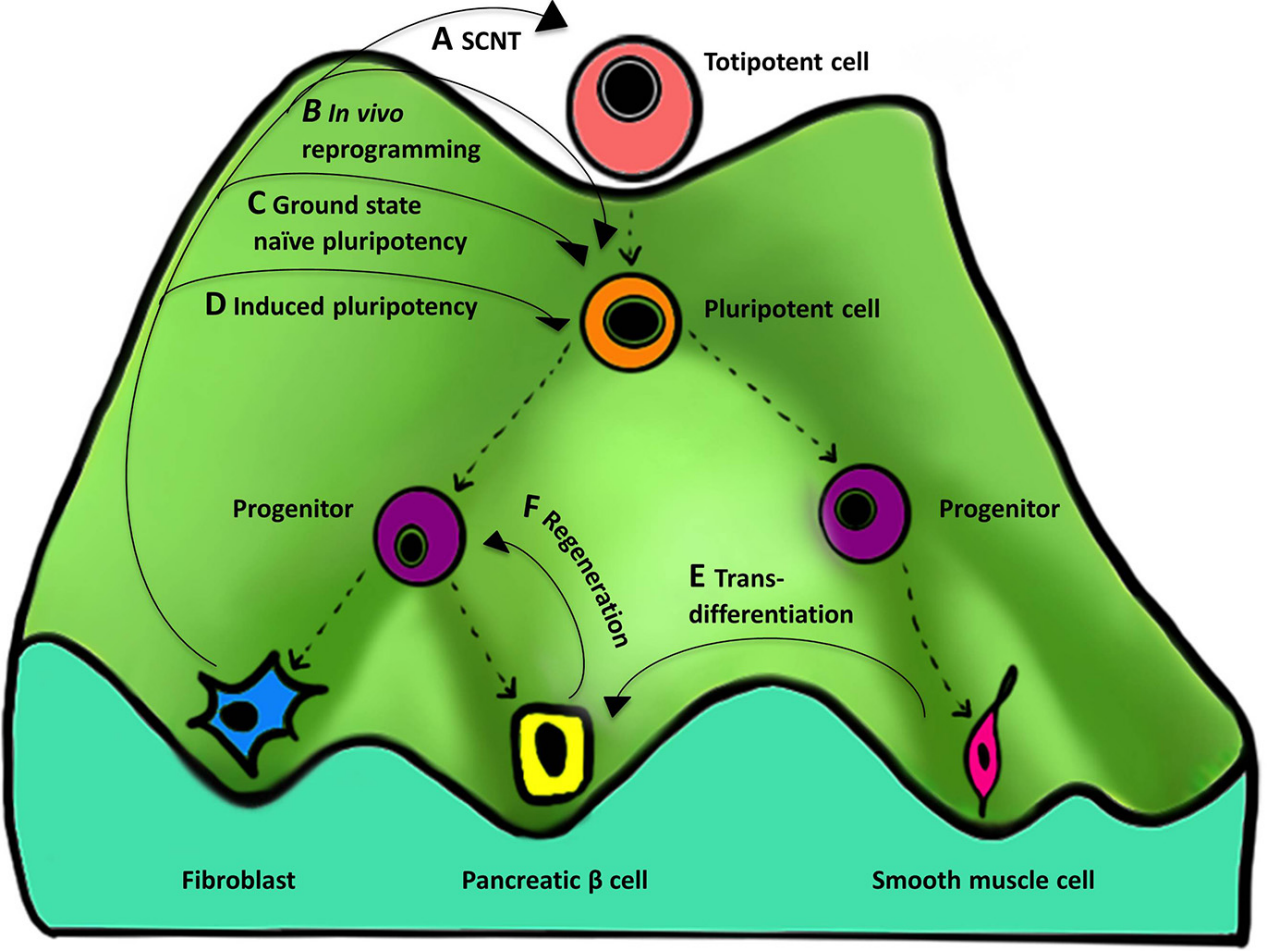
**Importance of the “epigenetic landscape” in cellular reprogramming**. An epigenetic landscape represents the process of cell fate decisions during development and is a graphical rendering of complex regulatory networks. The figure represents such an epigenetic landscape denoted by a “mountain” with its various “valleys.” The top of the mountain displays a totipotent cell that is completely undifferentiated and represents ultimate “stemness,” whereas at the bottom of the mountain are differentiated cells resulting from differentiation of their predecessors. Proceeding from the top of the landscape to the bottom, the cell changes from undifferentiated (totipotent and pluripotent), to partially differentiated (progenitor), to terminally differentiated (eg., fibroblast). **(A–F)** represent the different methods and their course on an epigenetic landscape, showing reversal of cellular identity to a more primitive state. There are four types of methods that are currently known for reprogramming to **(A)** totipotency, **(B,C)** near totipotency, and **(D)** pluripotency. (For simplicity, we have excluded reprogramming methods using only small molecules, cell-extract treatment, and cell fusion that can also result in ESCs-like cell formation). **(A)** SCNT gives rise to a totipotent cell capable of forming a complete organism. **(B)** *In vivo* reprogramming using OSKM generates iPSCs that are additionally capable of contributing to the trophoectoderm lineage and express embryonic and extra-embryonic markers. **(C)** *In vitro* reprogramming to ground state naïve pluripotency using NHSM media generates iPSCs more similar to mouse naïve ESCs. **(D)** Induced pluripotency generates iPSCs similar to but less pluripotent than **(A–C)**. Other processes that also involve manipulation of the epigenetic regulatory networks include **(E)** transdifferentiation—one differentiated cell type (smooth muscle cell) is converted to another (pancreatic beta cell) and **(F)** regeneration—derivation of a progenitor-like cell from a differentiated cell during wound healing.

The role of epigenetics during cellular reprogramming can be shown figuratively as an “epigenetic landscape.” An epigenetic landscape graphically represents the process of cell fate decision during development. Such a landscape is in reality a product of complex gene networks that are epigenetically regulated but can be represented figuratively in the form of a “mountain” with numerous “valleys.” Cells (represented by balls) reside in the valleys (epigenetically stable networks) and a cell can go down any valley depending upon which stimuli it receives to attain a differentiated state (Figure 3). Such epigenetic landscapes and the related complex gene regulatory networks that regulate cell fate can be used to predict the TFs associated with particular cell fates (Lang et al., 2014).

Reprogramming cell fate works through the manipulation of networks governing an epigenetic state. Different types of reprogramming can result in producing undifferentiated cells with varying degree of “stemness” by moving the cell back to the top of the mountain. Four cellular reprogramming methods, namely SCNT, and induced pluripotency (*in vivo, in vitro, and in vitro* using a special media) are noteworthy and are described below.

The totipotent cell resides at the very top of the landscape, and currently SCNT is the only method capable of reprogramming a somatic cell to a totipotent cell capable of forming a complete organism (Figure 3A). Below this would be a near-totipotent stem cell that has been generated through one of the following two methods: *in vivo* reprogramming using OSKM that generates iPSCs that are additionally capable of contributing to the trophoectoderm lineage and express embryonic and extra-embryonic markers (Abad et al., 2013) (Figure 3B), or *in vitro* reprogramming to ground state naïve pluripotency using NHSM media (see Section Induced Pluripotency above) that generates iPSCs more similar to mouse naïve ESCs and demonstrates the potential to overcome problems related to epigenetic memory and lineage differentiation biases (Gafni et al., 2013) (Figure 3C). The conventional *in vitro* method of TF-based reprogramming generates iPSCs that are similar to ESCs but often have problems related to epigenetic memory and lack totipotent cell-like features (Papp and Plath, 2011; De Los Angeles and Daley, 2013) (Figure 3D).

Other processes that also involve manipulation of the epigenetic regulatory networks include transdifferentiation (Figure 3E) and regeneration (Figure 3F). In transdifferentiation one differentiated cell type is converted to another through direct reprogramming (Takahashi, 2012), by avoiding going all the way back up the mountain to an intermediate pluripotent state. Regeneration is similar in that it results in derivation of SSCs or progenitor-like cells from a differentiated cell during wound healing, for example the formation of a blastema (an undifferentiated cell mass) in amphibians (Gurtner et al., 2008) that involves the cell going half-way back up the mountain.

A new framework has been developed in order to elucidate the role of epigenetic landscapes in reprogramming that combines the techniques of whole genome expression profiling and spin glass physics (Lang et al., 2014). This model has verified that partially reprogrammed cells co-express TFs associated with multiple cell fates, has reproduced known reprogramming protocols, and has the potential to generate new reprogramming protocols to create novel cell types (Lang et al., 2014). Defining epigenetic landscapes can not only help to gain better insights into reprogramming processes, but also to design highly efficient protocols. This can be done by targeting epigenetic modifiers involved in the differentiation process at specific positions in the landscape, as well as identifying easy access points in the hierarchy at which reprogramming can be induced for high efficiency.

Now that reprogramming technology has been described in detail we will move on to practical applications.

## Modeling human disease *in vitro*

**Figure 4:**
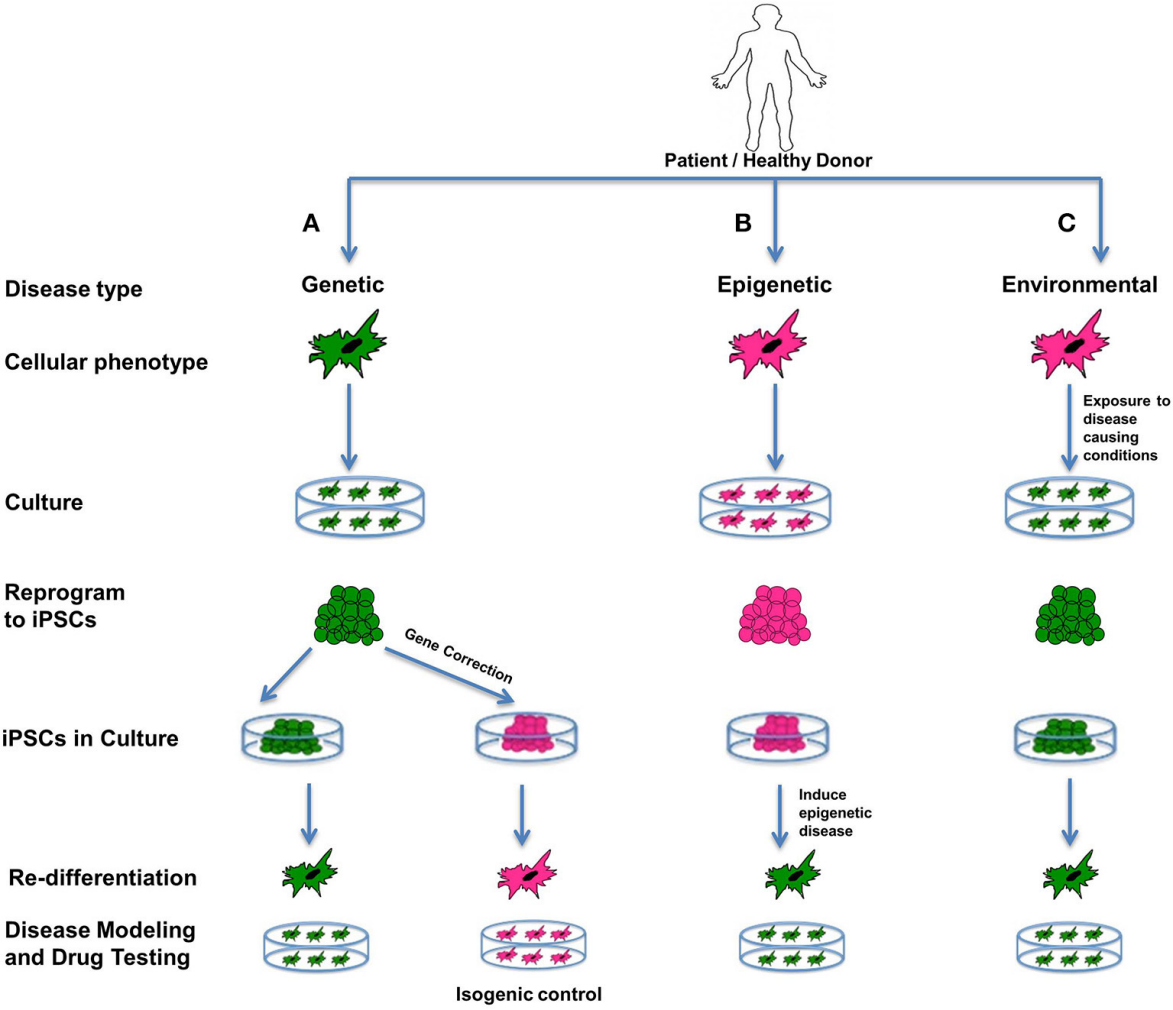
**Disease modeling**. iPSCs are an excellent source for modeling genetic, epigenetic, and environmental diseases. Such cellular models representing diseased phenotypes can be used for understanding the interplay between the genetics, epigenetics and environment involved in the disease, and can expose unknown details about disease pathophysiology, and can be used for screening drugs. In the figure all green cells represent diseased cells, and all pink cells represent healthy cells. **(A)** Genetic diseases can be modeled by reprogramming diseased cells to iPSCs and then re-differentiating them to produce a diseased phenotype. Additionally, these iPSCs can be corrected for the genetic mutation involved in the disease using gene-editing technology. On re-differentiation, corrected iPSCs produce healthy cells that can be used as isogenic controls. **(B)** Epigenetic diseases can be modeled using healthy cells that are reprogrammed to iPSCs and then induced toward an epigenetic disease state by recapitulating an environment containing the epigenetic factor(s) contributing to the disease. If iPSCs retain an epigenetic mark when in culture, or after being redifferentiated to the desired cell type, it indicates that the epigenetic mark is permanent and is likely to be passed on to offspring or carried by germ-line cells. It can also mean that the particular cell type is predisposed to retaining that epigenetic mark. Patient-specific models can be used as special models, as they can involve known epigenetic factors contributing to the disease. **(C)** Acute environmental diseases can be modeled using healthy cells by exposing them to a disease-causing environment that results in genetic damage or instability in the cells. For disease modeling, such cells can be reprogrammed to iPSCs and redifferentiated to diseased phenotypes. All of the above models can help us gain better insight into the diverse factors affecting a complex disease in terms of susceptibility, prognosis as well outcomes.

We can group human diseases into three broad categories: genetic, epigenetic and acute environmental (Cherry and Daley, 2012). Modeling of all three types is possible *in vitro* using stem cells, and an excellent way to study the intricate mechanisms and pathways underlying the etiology and pathophysiology of disease (Figure 4). Stem cells in general are ideal for creating “disease-in-a-dish” models because of their capacity for self-renewal and differentiation, their potential for recapitulating disease pathogenesis, and also their amenability for developing and testing therapeutics (Sterneckert et al., 2014).

The first and simplest types of diseases are monogenic disorders. For example Fanconi anemia (FA) has been studied using patient-derived, integration-free iPSCs with and without *in situ* gene correction (Liu et al., 2014). These FA-iPSCs are also useful for drug screening and shortlisting compounds that can trigger their hematopoietic differentiation potential (Liu et al., 2014) (Figure 4A). Likewise, iPSCs have been derived from cells obtained from patients suffering from a wide and rapidly-growing number of diseases such as Hutchinson-Gilford Progeria Syndrome (HGPS, see below), cystic fibrosis, Parkinson's disease, familial amyotrophic lateral sclerosis (ALS), dyskeratosis congenita, epilepsy, autism, spinal muscular atrophy, Huntington's disease, adenosine deaminase severe combined immunodeficiency, sickle cell anemia, long QT syndrome type I, glycogen storage disease type 1a, Alzheimer's disease, diabetes, and Down's syndrome and can be differentiated toward disease-specific lineages in order to study the genotype-phenotype interactions of the disease, as well as to screen new drugs that could be capable of reversing cellular pathology (Liu et al., 2011a; Unternaehrer and Daley, 2011; Cherry and Daley, 2012; Ess, 2013; Miller et al., 2013; Abdelalim et al., 2014; Acimovic et al., 2014; Hibaoui et al., 2014; Mohamet et al., 2014) (Figure 4A).

Monogenic diseases are easy to model in culture, but it is becoming increasingly clear that most diseases are a manifestation of altered genetics and/or epigenetics, with factors like age and environment complicating things further. It is necessary to consider such interlinked effects on different cell types in the body, especially in the case of late-onset disease, and for such purposes iPSCs can serve as an excellent model (Csobonyeiova et al., 2014). Thus, in addition to modeling monogenic diseases, cellular diseased phenotypes derived from iPSCs can be useful in studying complex diseases having genetic and epigenetic components, or having unknown sporadic genetic or acute environmental etiologies (Grskovic et al., 2011; Zhu et al., 2011). Such cellular phenotypes are also useful for screening drugs and understanding the pathophysiology of complex diseases (Grskovic et al., 2011).

Epigenetic diseases can be subtly contrasted with environmental diseases based on the type and extent of damage caused by the epigenetic or environmental factor. Specifically, when iPSCs are exposed to epigenetic factors, the iPSCs and redifferentiated cells may gain temporary or permanent epigenetic marks and manifest the diseased phenotype. Modeling epigenetic diseases in such a way can elucidate the trans-generational potential of the epigenetic factors i.e., whether the epigenetic mark is strong enough to pass on through the germ line to the next generation. Compared to epigenetic diseases, acute environmental insults do not cause heritable genetic or epigenetic effects, but rather the effect is seen at the somatic DNA sequence or protein level (Cherry and Daley, 2012). For example, an acute environmental disease like melanoma that is frequently caused by excessive sunburn (ultraviolet ray damage) can lead to direct DNA damage in the form of pyrimidine dimers (Anna et al., 2007). Such sporadic environmental insults, including spontaneous deaminations, change the DNA sequence as well as alter the cellular biochemistry through upregulation of DNA repair processes, alter expression of cell cycle regulators, or possibly cause apoptosis (Fulda et al., 2010).

Using patient-specific iPSCs we can recapitulate the suspected effects of the environmental or epigenetic component known to have contributed to the patient's disease in order to understand its severity or mechanism of action before, during, or after reprogramming (Figures 4B,C). Cells from any source can be epigenetically manipulated to mimic epigenetic (Figure 4B) or acute environmental (Figure 4C) alterations contributing to disease. Such models can help us gain better insight into the environmental or epigenetic factors affecting a complex disease in terms of susceptibility, prognosis as well as outcomes. Patient-specific models can also be used as special models, as they can involve known epigenetic changes contributing to the disease.

iPSCs can also be used for modeling disease at the organ level as well as understanding systemic diseases. For example human iPSCs can be used for *in vitro* development of complex structures like the retina by spatiotemporally recapitulating the *in vivo* steps of retina formation and making it possible to test future therapies *in vitro* (Zhong et al., 2014).

## Treating human disease

Beyond modeling disease, reprogramming can also be used to treat disease. While the technology of cellular reprogramming has been developed and refined there have also been significant ongoing developments in other complementary technologies such as gene editing, progenitor cell production, and tissue engineering that we will describe below. These are converging to a point that will allow us to treat almost any disease. Personalized medicine may soon become a reality, with cellular harvest and reprogramming, genetic engineering and tissue engineering becoming a routine procedure. These technologies are the foundations of what is becoming a fully-functional field of regenerative medicine.

### *In vitro* reprogramming and complementary technologies

#### Induced pluripotent stem cells

**Figure 5:**
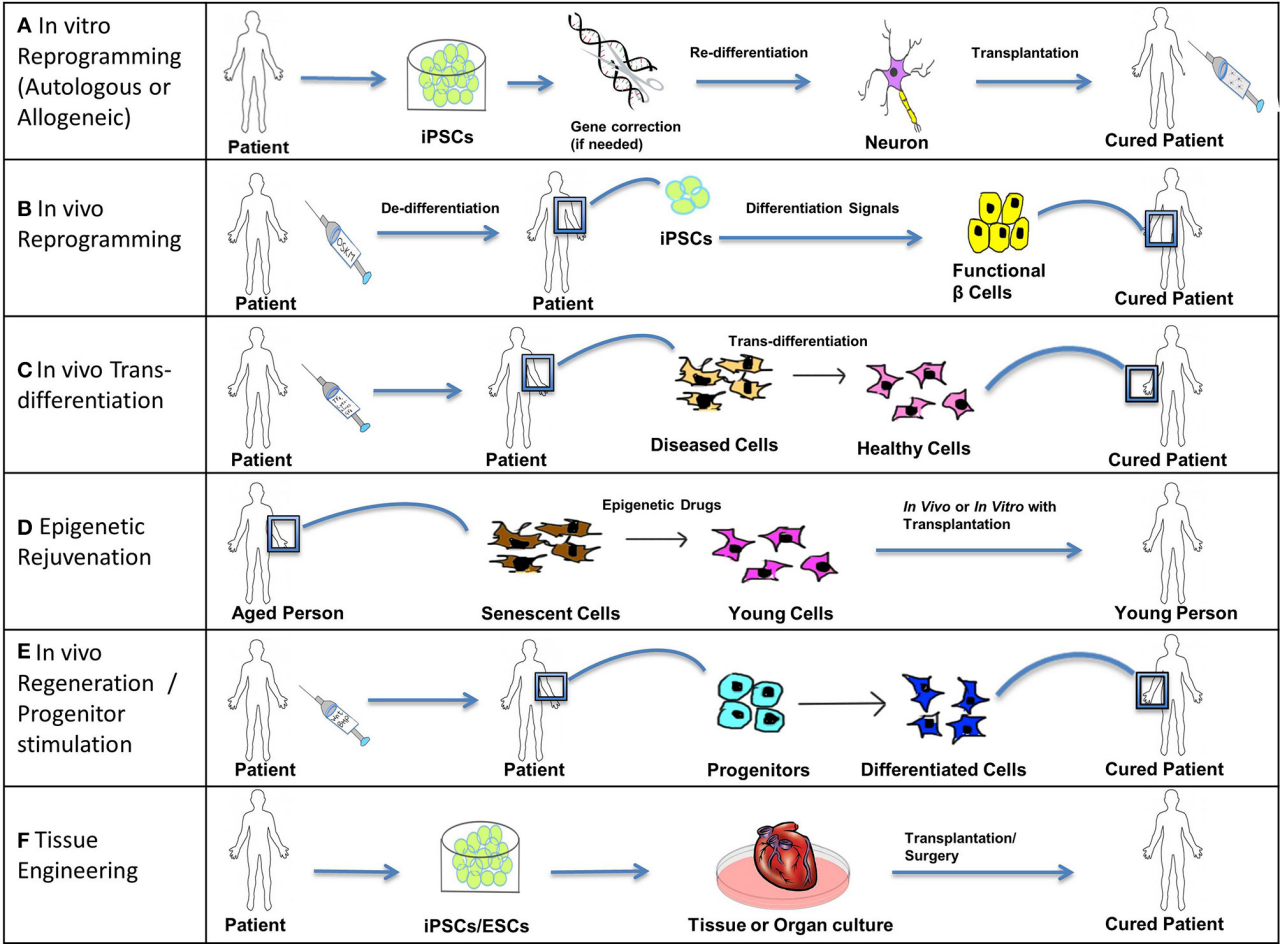
**Treating disease**. Stem cells (iPSCs, ESCs, and SSCs/Progenitors) can be used in the following ways to treat acute, chronic, and degenerative diseases. **(A)** *In vitro* reprogramming. iPSCs reprogrammed *in vitro* using different combinations of transcription factors can be further differentiated to specific lineages and these cells can be transplanted into the patient suffering from a degenerative disease or injury. For example, autologous iPSCs generated from fibroblasts of a patient suffering from Parkinson's disease can be differentiated into functional dopamine secreting neurons and can be transplanted into the patient's brain through surgery. In cases where solely transplanting functional cells is inadequate to completely cure a genetic disease, a permanent fix of the genetic mutation involved is required. Thus, an additional step of gene correction can be performed on the patient-specific iPSCs using gene-editing technologies like CRISPR/Cas. Allogeneic iPSCs might be useful to treat traumatic acute injuries like spinal cord injury, as they would be available immediately when required. **(B)** *In vivo* reprogramming. iPSCs can be generated *in vivo* at the site of non-functional or damaged tissue (by administration of reprogramming factors like OSKM) and can then be differentiated toward a specific lineage (by the administration of differentiation signals). For example, iPSCs generated *in vivo* from pancreatic cells of a patient suffering from diabetes can be differentiated into functional β cells secreting insulin and thus restore the lost function by repopulating the pancreatic tissue with functional β cells. **(C)** *In vivo* trans-differentiation. A somatic cell can be trans-differentiated *in vivo* into a different type of somatic cell using transcription factors, growth factors, etc. such that the new cell type helps to restore the lost function of the diseased or damaged tissue. This process is noteworthy as it avoids the intermediate step of reprogramming somatic cells to pluripotency; however this process is not yet well-established. **(D)** Epigenetic rejuvenation. The idea behind epigenetic rejuvenation is that epigenetic drugs can be used to convert senescent cells to young cells such that aging is reversed without affecting the differentiation or specialized function of the cell. This can be done *in vivo* directly or *in vitro* followed by transplantation of the rejuvenated cells into the patient. **(E)** *In vivo* regeneration/progenitor cell stimulation. Progenitors can be induced to differentiate into their successors by injecting differentiation signals like Wnt and Bmp proteins in order to achieve regeneration *in vivo*, which will restore lost function of damaged or diseased tissue. This process avoids procedures involving induction of pluripotency as well as transplantation into the patient. **(F)** Tissue Engineering. Patient-specific iPSCs (with optional gene correction step) or ESCs can be used to culture tissues and organs *in vitro* using specialized scaffolds and organ molds or using bioprinting. Such cultured organs can then be transplanted into a patient through advanced surgical procedures to restore the function of an entire organ or organs, in the case of chronic or systemic diseases, as well as to treat acute illnesses like myocardial infarction with cardiac tissue transplants.

The greatest advantage of using *in vitro* reprogramming is that we can generate patient-specific iPSCs (autologous) and eliminate the threat of rejection, or have well-characterized iPSC cell-lines (allogenic). Reprogramming *in vitro* is often more useful when used with complementary technologies like gene editing to treat genetic diseases by fixing the causative mutation and then repopulating damaged or non-functional tissue with the desired healthy cell type (Figure 5A).

Generation of iPSCs enables *in vitro* coordination of differentiation into cells of all three germ layers (endoderm, mesoderm, and ectoderm). Where the aim is simply to repopulate the non-functional or damaged tissue with functional cells, transplantation with iPSC-derived cells could cure the patient. For example, mouse embryonic fibroblasts or mouse pancreas-derived epithelial cells reprogrammed to iPSCs and then differentiated to functional pancreatic beta cells were capable of treating diabetes in mice (Jeon et al., 2012). These iPSC-derived beta-like cells released insulin and on transplantation into non-obese mice, normalized the blood-sugar level (Jeon et al., 2012). If treating a disease involved fixing a genetic mutation permanently, then gene-editing technologies (which are explained in detail in section Gene editing using CRISPR/Cas9, ZFN, TALENs etc) can be used as an additional step before differentiating the iPSCs into the desired cell type. For example in mice, the correction of a mutation in iPSCs by gene therapy using homologous recombination, followed by autologous transplantation back into the mouse, has led to the replacement of diseased cells and rejuvenation of functional tissue in single-gene defects like sickle cell anemia. Such procedures have overcome problems associated with immune rejection and validated the role of iPSCs in regenerative medicine (Hanna et al., 2007; Takahashi and Yamanaka, 2013). However, autologous iPSCs will incur a comparatively higher medical cost compared to allogenic ones, and their creation could take a longer time compared to allogenic iPSCs. Also they might not be available immediately when required for the treatment of acute injuries like spinal cord injury, implying the need for HLA-matched donor allogenic iPSCs (Takahashi and Yamanaka, 2013), unless they can be prepared beforehand.

One of the most promising cell types derived from iPSCs are neural cells (Svendsen, 2013). Neurons derived from iPSCs demonstrate great promise in revitalizing and repopulating the central nervous system, and have promise for use in the development of neurologically based disease models such as Huntington's and Parkinson's diseases (Kriks et al., 2011; Liu et al., 2013b).

Potential risks associated with clinical iPSC applications include the possibility that viral or other agents used for reprogramming could trigger harmful immune or inflammatory responses (Zhao et al., 2011; Guha et al., 2013). The prevention of vector or transgene integration into the host genome or residual transgene expression once reprogramming has been achieved is important in order to avoid such unwanted responses. This problem has now been resolved with the use of non-integrating episomal vectors to create integration-free iPSCs (Hu et al., 2011; Su et al., 2013). The use of non-viral vectors such as the transposon-based systems like Sleeping Beauty (SB) and piggyBac (PB) offer a cost-effective and safe alternative (Talluri et al., 2014). Several experiments to eliminate the use of more than one or two reprogramming factors during induced pluripotency have also been conducted (Radzisheuskaya and Silva, 2014). For example easily accessible dermal papilla cells (specialized mesenchymal cells endogenously expressing Sox2, Klf4, Myc) from the hair follicles of skin have been reprogrammed with Oct4 alone and without any other chemical compounds or small molecules (Tsai et al., 2011).

#### Gene editing using CRISPR/Cas9, ZFN, TALENs etc

As stated, the use of the combined techniques of reprogramming and genetic engineering followed by cellular therapy can potentially correct a genetic defect and repopulate a degenerated or damaged tissue with the required cell type and cure a genetic disease. Such technology can also be used to manipulate and study model organisms as well (Gaj et al., 2013).

Gene editing technologies are methods used to fix an endogenous mutation, or to make a gene inactive for the purpose of repairing or deleting a mutation. Recent progress in gene editing technology has made it possible to induce a site-specific DNA cleavage followed by repair. This results in high precision genome editing in cultured cells and whole organisms; useful in designing therapeutic treatments and creating models *in vitro* and *in vivo* (Kim and Kim, 2014).

Gene editing technology is based on the use of RNA sequences or proteins that bind to specific DNA sequences and recruit nucleases to introduce double strand breaks in the target sequence. Once DNA damage occurs, homologous or non-homologous end joining processes repair the DNA sequence. With homologous recombination, the wrong nucleotides/s can be replaced with the correct ones to repair a genetic mutation. Current precise gene editing methods include the following three types depending on the type of repair and nucleases involved: 1. Zinc finger nucleases (ZFN), 2. Transcription activator-like effector nuclease (TALEN), and 3. Clustered regularly interspaced short palindromic repeats (CRISPR).

1. ZFN- This is the most commonly used type at present, and is comprised of a eukaryotic zinc finger binding protein domain coupled with a nuclease.
2. TALEN- This type similarly uses a transcription activator-like effector (TALE) domain coupled with a nuclease.
  ZNFs and TALENs include programmable, sequence specific DNA binding modules linked to a non-specific DNA cleavage domain and are capable of introducing a variety of genetic modifications (Gaj et al., 2013).
3. CRISPR- This is the newest type of gene editing technology based on RNA interference and uses CRISPR and CRISPR associated genes (Cas) from bacterial and archeal immune systems. The CRISPR loci have short palindromic repeats (24–37 base pairs) separated by unique spacer sequences of equal length that can integrate foreign DNA, [which in the source bacteria/archea can be used as a type of memory in the case of a future attack by the same foreign DNA (Richter et al., 2013)]. When a CRISPR sequence is transcribed, the foreign DNA is transcribed along with it. The processed RNA in the form of crRNA and tracrRNA is responsible for sequence specific silencing by recognizing the target foreign DNA sequence and degrading it with Cas proteins that have nuclease activity (Gaj et al., 2013).
  This RNA-guided genome editing tool could be used to correct disease- associated genes in zygotes and human cell lines (High et al., 2014). The CRISPR/Cas system can effectively replace the traditional method of producing mutant mice by sequential recombination since this technology allows a highly efficient single-step approach for production of ES cells with mutations in multiple genes, as well as generation of mutant mice by direct embryo manipulation (Wang et al., 2013).
  CRISPR- based genome editing is also useful in disease modeling because it is very efficient at generating allelic isogenic models to study diseases *in vitro*. For example the combination of CRISPR technology and an antibody-based method to screen recombination events has been used to generate genetically modified human cell lines for the study of Huntington's disease (An et al., 2014). The Type II CRISPR/Cas9 system is gaining special recognition for its ability to accelerate the production of cell lines, especially in combination with reprogramming, and the production of animal models with a desired genetic makeup for drug screening and pre-clinical evaluation. Also it seems to be a desirable candidate for gene therapy alone (Yingze Zhao, 2014).

### *In vivo* reprogramming

Other potential ways of using iPSCs for treating diseases would be by generating them *in vivo*. These *in vivo* iPSCs can further generate desired cell types in the presence of appropriate signals for re-differentiation (Figure 5B). As described in section Induced Pluripotency and Epigenetics during cellular reprogramming, *in vivo* transitory induction of OSKM in mice led to *in situ* reprogramming and gave rise to iPSCs from a variety of cell types (Abad et al., 2013). These iPSCs represented totipotent-like features and they resembled ESCs more closely compared to *in vitro* generated iPSCs (Abad et al., 2013). Another *in vivo* study showed the generation of induced adult neuroblasts (iANBs) from resident astrocytes using a single TF Sox2 and these iANBs formed mature neurons when targeted for differentiation using noggin, BDNF or histone deacetylases inhibitor (Niu et al., 2013).

### *In vivo* trans-differentiation

Transdifferentiation is the process in which a particular somatic cell is switched from one lineage-specific identity to a completely different identity (Graf, 2011; Vierbuchen and Wernig, 2012); in other words, the direct conversion of one type of somatic cell into another type, bypassing the intermediate step of dedifferentiation (Figure 5C). As described in Section Transdifferentiation/direct reprogramming transdifferentiation *in vivo* converted adult murine pancreatic exocrine cells to β-cell like population using the transcription factors Ngn3, Pdx1, and Mafa (Zhou et al., 2008). Another example is the *in vivo* transdifferentiation of human peripheral blood CD34^+^ cells into non-hematopoietic lineages such as cardiomyocytes (Yeh et al., 2003). However, it is important to note that such processes occur only under the conditions of severe tissue damage (Yeh et al., 2003).

### Epigenetic rejuvenation

Epigenetic rejuvenation is the concept of erasing an “aged” epigenome to obtain an epigenome that corresponds to the one seen in a “young” healthy cell along with maintaining its differentiated state and rejuvenating its function (Manukyan and Singh, 2012). The idea behind epigenetic rejuvenation is to treat aged cells from patients *in vitro* and transplant them back into the tissue and replace aged or unhealthy, damaged cells (Manukyan and Singh, 2012) (Figure 5D). (Please refer to Section Hutchinson-Gilford Progeria Syndrome for additional details.)

### *In vivo* regeneration/progenitor cell stimulation

*In vivo* regeneration involves the repair of tissue damage by stimulation of endogenous stem cells (SSCs/progenitors) by growth factors, cytokines, transcription factors, and second messengers (Figure 5E). A promising strategy to treat degenerative and chronic disorders resulting from loss of function of a particular type of cell, is through harnessing the intrinsic regenerative potential of the progenitor cells which are responsible for creating the specific type of damaged cell and restoring their lost function. Studies involving the use of regenerative factors like developmental signals that play a role in the regeneration of a damaged tissue, suggest that the latent/dormant intrinsic potential can be triggered to harness the *in vivo* regeneration capacity in a disease or trauma-struck individual (Figure 5E). Such changes during regeneration are accompanied by epigenetic modifications. A few examples of signaling pathways and differentiation factors, which are being studied and could potentially activate regeneration (thus should be considered as suitable targets) are described below.

1. Wnt, a ligand working in conjunction with a receptor, involved in the transcription of β- catenin promotes morphogenesis and has the capacity to initiate the regeneration of broken bone to treat bone deformities or injuries, as well as cause Muller progenitors to differentiate into retinal progenitors, thereby ameliorating retinal trauma, and possibly blindness (Minear et al., 2010; Liu et al., 2013a).
2. Platelet-derived growth factor (PDGF) and insulin-like growth factor (IGF) have been shown to stimulate the regenerative capacity of mammalian skeletal and neural tissue (Rutherford et al., 1992; Mason et al., 2000).
3. Bone morphogenic protein (BMP) is essential for ESC renewal whereas basic Fibroblast growth factor (bFGF) increases the differentiation potential of MSCs into chondrogenic, osteogenic and adipogenic lineages and epidermal growth factor receptor (EGFR) increases the proliferative potential and survival capacity of MSCs (Bhattacharyya et al., 2012).
4. Lin28 is expressed during embryogenesis as it plays an important role in pluripotency and development, also its overexpression promotes epidermal hair regrowth, and digit repair in mice by altering the bioenergetic state during tissue repair following enhanced translation of metabolic enzymes that increase glycolysis and oxidative phosphorylation (Shyh-Chang et al., 2013).
5. The transient inactivation of two tumor suppressors ARF (alternative reading frame protein) and RB (retinoblastoma protein) in mammalian cells resulted in dedifferentiation restoring regenerative capacity and produced differentiated cell types when cultured in the presence of proper signaling factors suggesting that terminally differentiated mammalian cells can be induced for regeneration without neoplastic transformation (Pajcini et al., 2010).
6. Hox genes play an important role in embryonic pattern formation and can be programmed such that their previous epigenetic memory is erased resulting in plasticity, as observed in iPSCs, that can in turn be used to trigger regenerative capability of injured tissue (Wang et al., 2009).

### Tissue engineering

Tissue engineering is the field of engineering tissues, or organs grown from *in vitro* cultures of stem cell-derived differentiated cells, followed by their transplantation (Figure 5F).

Tissue engineering is the *de novo* production of tissues or organs using starting material like cells, extracellular matrix and scaffolds for supporting the three dimensional process of tissue formation (Fisher and Mauck, 2013). This technology has been translated into actual therapies for skin replacement and cartilage repair (Berthiaume et al., 2011). The first step in tissue engineering is to procure the desired cell types required for constructing the tissue or organ. iPSCs as well ESCs serve as a good source for producing the cells through directed differentiation. For example, iPSC-derived multipotent neural crest stem cells (NCSCs) have been used to engineer nerve conduits by seeding NCSCs into nanofibrous tubular scaffolds and such nerve conduits were used for regenerating sciatic nerve (Wang et al., 2011). Tissue engineering uses conventional methods for scaffold fabrication that are probably inadequate (Seol et al., 2014), but the latest bioprinting technology can construct 2-D and 3-D tissues and organs using additive manufacturing technology that allows precise placement of cells, biomaterial and biomolecules in an adequately fabricated scaffold (Seol et al., 2014).

### Clinical trials for stem cell therapy in humans

The U.S. Food and Drug Administration defines somatic cell therapy as the administration to humans of autologous, allogeneic or xenogeneic living non-germ line cells, other than transfusion blood products, which have been manipulated, processed, propagated or expanded *ex vivo*, or are drug-treated (Health et al., 2001; Parson, 2006; Bieback et al., 2011; George, 2011).

With the FDA approving the first ESCs Phase I clinical trials for acute spinal cord injury in 2011, the science behind stem cell biology and regenerative medicine made its way to practical applications in the real world (Table 1). Extensive efforts from the California Institute of Regenerative Medicine (CIRM), National institute of Health (NIH) and other organizations around the world has allowed stem cell therapies to progress into clinical trials for a variety of diseases, many of which have made significant progress. Clinical trials using mesenchymal stem cells (MSCs) for treating bone and cartilage diseases, neurodegenerative diseases, heart diseases, gastrointestinal diseases, autoimmune/immune rejection diseases, and cancer have shown a lot of progress with massive funding from CIRM, their collaborating partners and others (Trounson et al., 2011) (Table 1).

**Table 1:** **Resources for tracking progress in the Field of Regenerative Medicine**.

**Sr. No**.	**Topic**	**Affiliation**	**Access data**
1.	Current status of therapies undergoing clinical trials	CIRM	http://www.cirm.ca.gov/sites/default/files/files/about_stemcells/Portfolio%20table_10-13.pdf
2.	Publically and Privately supported Clinical studies around the world	NIH	http://clinicaltrials.gov/ct2/home
3.	Clinical trials and stem cell treatment	EuroStemCell	http://www.eurostemcell.org/clinical-trials
4.	Adult stem cell clinical trials	Stem cell research facts	http://www.stemcellresearchfacts.org
5.	Disease information	CIRM	http://www.cirm.ca.gov/our-progress/disease-information
6.	Current status of funding and research	CIRM	http://www.cirm.ca.gov/sites/default/files/files/funding_page/Portfolio%20summary%2006-03.pdf

The most advanced clinical trials listed by EuroStemCell include testing of therapies for bone, skin, corneal disease and injury, the majority of which use mesenchymal stem cells. The NIH lists two active clinical trials for the eyes diseases Age related Macular Degeneration (AMD) and Stargardt's muscular dystrophy using cells derived from human ESCs, being produced by Advanced Cell Technology. Other sources like bone marrow stem cells for treating cancer and blood disorders, human central nervous system stem cells for AMD and human spinal cord stem cells for ALS are also in clinical trials. Human MSCs are being used in clinical trials by Osiris therapeutics for Type 1 diabetes, cardiomyopathies, Graft vs. host disease and Crohn's disease. More details about these ongoing clinical trials can be found through the links for online resources provided in Table 1.

## Candidate diseases for treatment using reprogramming

In this section, we provide specific examples of how reprogramming and complementary technologies can be used to treat and eventually cure human disease.

### Aging and age-related diseases

A few decades ago, Richard Cutler proposed that aging was a result of cells drifting away from their proper state of differentiation due to changes in gene expression, through a process he termed “dysdifferentiation” (Kator et al., 1985). This process could be reinterpreted today as epigenetic drift. These changes are so predictable that recently an “epigenetic clock” has been formulated that can very accurately predict the age of an individual based solely on the methylation of specific genes in genomic DNA (Horvath, 2013). Indeed, epigenetic changes represent a major aspect of aging, and epigenetic manipulation may allow reversal of these damaging effects (Campisi and Vijg, 2009; Munoz-Najar and Sedivy, 2011).

It has been proposed that if we can reverse the epigenetic changes associated with aging, we could actually reverse aging itself. This epigenetic approach to reversing aging has been termed “epigenetic rejuvenation” (see Section Epigenetic Rejuvenation and Figure 5D). Although the very existence of animals cloned by SCNT proves that epigenetic rejuvenation is possible in principle, only recently have *in vitro* experiments on human cells shown that this is possible. Indeed, even centenarian fibroblasts can be rejuvenated, and young fibroblasts cultured to senescence can be reprogrammed to a pluripotent state (Lapasset et al., 2011; Villeda et al., 2011; Loffredo et al., 2013). Recently, very detailed experiments have shown that age-related and tissue-specific DNA methylation patterns remain erased in mesenchymal stem cells produced by induced pluripotency (Frobel et al., 2014).

Epigenetic reversal also crucially includes reactivation of telomerase expression, which resets the chromosome “aging clock” back to a young state. Reprogramming “old” cells to “young” cells will eventually enable the repopulation of an aged and/or degenerated tissue with cells capable of extended proliferation. Perhaps this strategy could eventually be applied to the whole body.

An example of a type of epigenetic rejuvenation already practically applied is heterochronic parabiosis: experiments with two animals of a different age joined together surgically, showed that tissue-specific stem cells from an aged animal could be rejuvenated by exposing them to a “young” environment (Rando and Chang, 2012; Conboy et al., 2013). This “young” environment involves factors such as chemokine's, cytokines and rejuvenating Wnt and TGFβ signaling pathways (Conboy et al., 2013). On the other hand “young” stem cells adopt a more aged structure and function (Conboy et al., 2005). Unlike reprogramming, heterochronic parabiosis maintains the differentiated states of the cells and rejuvenates the regenerative potential of old cells in the milieu of a young environment, thus uncoupling dedifferentiation and rejuvenation (Conboy et al., 2013). This type of rejuvenation might have an epigenetic basis regulated by a switch that could possibly convert a cell from “old” to “young” or vice versa.

Aging also involves mitochondrial dysfunction as well as defective mitochondrial DNA. SCNT can be used to correct diseases caused by defective mitochondrial DNA (mtDNA) because in NT-ESCs the mtDNA exclusively comes from the oocyte, and only the nucleus from the donor/patient, thus making it possible to avoid defective mtDNA being carried over from the donor/patient (Tachibana et al., 2013). Surprisingly, induced pluripotency rejuvenates mitochondira, but in a different way to SCNT (Suhr et al., 2010).

#### Hutchinson-Gilford Progeria Syndrome

Hutchinson-Gilford Progeria Syndrome (HGPS) is a segmental premature aging disease characterized by symptoms of premature aging, with a life expectancy in the early teens or twenties. The single point mutation responsible for causing this disease occurs in position 1824 (cytosine is replaced with thymine) of the Lamin A (*LMNA*) gene on chromosome 1. Lamin A is an intermediate filament protein that stabilizes the inner membrane of the nuclear envelope. The mutation results in a defective Lamin A that produces an abnormal protein called Progerin. Ultimately, this results in abnormally shaped, dysfunctional nuclei that in turn disrupt cell division and other cellular functions (Eriksson et al., 2003; McClintock et al., 2007; Korf, 2008).

Somatic cells from Progeria patients can be reprogrammed to iPSCs and the LMNA mutation can be corrected using gene-editing technology (see above). The fact that ESCs and iPSCs do not express *LMNA* (Constantinescu et al., 2006), allows the efficient correction of the mutation in iPSCs, and any disease-related pathology can be avoided in iPSC-derived differentiated cells. Notably, HGPS iPSCs exhibit downregulation of lamin A/C and Progerin, causing a lack of progeroid features in the iPSC population, rendering these iPSCs normal with respect to nuclear morphology, pluripotency, and epigenetic profiles (Liu et al., 2011a). HGPS iPSCs corrected for the *LMNA* mutation can then be differentiated into normal somatic cells (Liu et al., 2011b). These iPSC-derived differentiated cells could eventually be transplanted into patients using cellular therapy to repopulate the aged tissue with healthy proliferative cells free of the original Progeria mutation.

#### Age-related macular degeneration

Age-related macular degeneration (AMD) is a complex multifactorial diseases caused by the degeneration of the photoreceptors and retinal pigment epithelium (RPE) of the eye (Yonekawa and Kim, 2014). It is the most common cause of visual impairment in the elderly (+60 years) and it is broadly classified into two clinical categories, namely, the wet form (neovascular or exudative) and dry form (Cook et al., 2008). AMD, which is primarily known to be an age-related disease, is also affected by other risk factors like genetics, patient history, smoking, trauma, etc (Chakravarthy et al., 2010). Data from pre-clinical studies suggests that transplantation with ESCs-derived RPE or patient-specific iPSC-derived RPE (with correction of the gene mutation) may prevent photoreceptor degeneration in animal models of RPE degeneration (Schwartz et al., 2012; Heller and Martin, 2014). With the advent of techniques in *in vivo* reprogramming, another promising approach to cure AMD is to target the intrinsic potential of neural progenitors in a patient and drive their differentiation into RPE through the administration of appropriate differentiation signals. It has already been suggested that retinal stem cells (RSCs) that are multipotent, rare cells in the pigmented ciliary epithelium of peripheral retina can be enriched an directed to differentiate into photoreceptors using factors influencing neural retinal development (Ballios et al., 2012). Specialized knowledge of such differentiation and proliferation signals with the development of advanced techniques toward delivery and transplantation of stem cells or RPE lead us one step closer to the development of clinical therapies for AMD.

### Epigenetic diseases

Epigenetic diseases are caused by altered expression of genes caused by abnormal changes in the epigenetic profile of a cell. Reprogramming can be used to reset the epigenetic profile of any cell so that the cell becomes pluripotent. It can then be differentiated to obtain the cell type with a desired epigenetic profile. Such procedures followed by cellular therapy might be a potential treatment for epigenetic diseases caused by known epigenetic factors. Let us consider the example of polycystic ovary syndrome (PCOS) as an epigenetic disease.

PCOS is a heterogeneous endocrine disorder responsible for a large proportion of female subfertility (Azziz et al., 2004). The hallmarks of the disease are anovulation, excessive androgenic hormones, and insulin resistance. The PCOS phenotype is characterized by hypersecretion of both luteinizing hormone as a result of compromised hypothalamic sensitivity, and insulin in response to increased abdominal adiposity (Escobar-Morreale et al., 2005). Familial clustering of cases and the heritability of endocrine and metabolic aspects of PCOS indicate a dominant regulatory gene with incomplete penetrance and strongly indicate a genetic etiology (Diamanti-Kandarakis et al., 2006).

As described in Section Epigenetics above, environmental factors can induce epigenetic alterations, which potentially involve trans-generational inheritance of methylation patterns. The exposure of female rats to a mixture of pesticides showed that the first and third generation offspring were afflicted with PCOS (Manikkam et al., 2012). This demonstrated that PCOS might have an epigenetic basis caused by a differential methylation pattern in the diseased offspring. In humans aberrant gene methylation has been reported in patients with PCOS, suggesting a role for a bZIP TF, CEBPB, in insulin resistance and regulation of the interleukin-1 response element in the IL-6 gene and other genes important for the immune and inflammatory components of this disease (Natsuka et al., 1992; Harries et al., 2012; Shen et al., 2013). Inappropriate epigenetic reprogramming is a contributing factor in PCOS. Epigenetic alterations of peroxisome proliferator-activated receptor gamma 1 (PPARG1), nuclear co-repressor 1 (NCOR1), and histone deacetylase 3 (HDAC3) genes in granulosa cells are induced by hyperandrogenism (Qu et al., 2012).

Recently, PCOS-derived hESCs have been successfully isolated from the inner cell masses of blastocysts. These hESCs retain pluripotency, which allows the dissection of the *in vitro* pathogenesis of the disease. Eventually cell therapies will be developed for this disease.

### Combined genetic and epigenetic diseases

Some diseases long known to have genetic causes are now thought to also have epigenetic bases. Just as with epigenetic diseases, such diseases can be treated using reprogramming to re-establish the normal epigenetic profile but with the addition of gene correction. One of the most well-known such diseases is cancer.

#### Cancer

Cancer remains the leading cause of death worldwide with over 7.6 million deaths (13%) in 2008 (Jemal et al., 2011). Cancer does of course have a partly genetic basis: most human cancers are caused by gene mutations in one or more components of the p53 and/or the pRB pathway (Campisi, 2001). And most tumor suppressor genes can be classified into two types; Caretaker tumor suppressors which prevent DNA damage and Gatekeeper tumor suppressors which eliminate potential neoplastic cells via apoptosis (Campisi, 2005).

But besides the genetic mutations, cancer is also an epigenetic disease. The dysdifferentiation hypothesis of aging and cancer emphasizes the importance of instability of the differentiated state of cells due to improper gene regulation in the development of cancer (Zs-Nagy et al., 1988). Cancer is comprised of the following eight hallmarks, some of which may have a wholly epigenetic etiology: sustained proliferative signaling, evading growth suppressors, enabling replicative immortality, inducing angiogenesis, resisting cell death, activating invasion and metastasis, reprogramming of energy metabolism and evading immune destruction. (Hanahan and Weinberg, 2011). Reprogramming of metabolic pathways contributes to the development of the disease, which is evidently seen in cancer with upregulation of oxidative phosphorylation and glycolysis-associated tarnscriptomes (Lisanti et al., 2014). For example, cancer-associated fibroblasts found in tumor microenvironments have a distinctive energy metabolism reprogramming phenotype responsible for proliferation, migration, invasion and epithelial-mesenchymal transition (Tang et al., 2014).

Cancer cells have altered DNA methylation profiles such that the overall methylated DNA is less than that seen in normal cells. A study testing epigenetics as a molecular marker system for cancer noted 12 genes to be associated with promoter hypermethylation causing silencing of their respective genes. These were functional in cell cycle regulation, tumor suppression, DNA repair, metastatic potential and apoptosis and have been linked to 15 major tumor types, making epigenetics a significant tool in cancer detection and typing (Esteller et al., 2001). Epigenetic changes in cancer are becoming well-noted and epigenetic drugs like DNA methylation inhibitors and histone deacetylase inhibitors have shown promising results in clinical trials and are less toxic than the conventional chemotherapy drugs (Chen et al., 2014). Epigenetic modulators may be used to inhibit self-renewal and survival of quiescent cancer stem cells (CSCs) by making them sensitive to targeted or conventional chemotherapy (Li and Bhatia, 2011).

#### Other diseases

Genetic factors contributing to epigenetic variation like DNA sequence variants, differential expressions of genes that regulate chromatin remodeling and DNA methylation, can contribute toward an epigenetic etiology of a disease (Bjornsson et al., 2004). Familial forms of neurodegenerative diseases like Alzheimer's disease, Huntington's disease, Parkinson's disease, and ALS are known to have a genetic basis but increasing evidence points toward epigenetic alterations like DNA methylation, histone tail modifications, and RNA mediated mechanisms in sporadic forms (Kleivi, 2006).

Some autoimmune diseases such as systemic lupus erythematous, rheumatoid arthritis, multiple sclerosis, and type 1 diabetes mellitus suggest the contribution of environment to a genetically pre-disposed trait (Hewagama and Richardson, 2009). For example, Immunodeficiency chromosomal-instability facial anomalies syndrome (ICF) is one such disease, which is caused by mutation in the gene for DNA methyl transferase (DNMT3B) that further leads to abnormal methylation causing varied gene expression (Ehrlich, 2003; Bjornsson et al., 2004; Rodenhiser and Mann, 2006).

Other examples include the downregulation of the Reelin gene (RELN) in schizophrenia which leads to the reduction of RELN protein by 50% compared to a normal brain and this has been linked to an epigenetic modulation of the promoter of the RELN gene through increased expression of DNMT1 (Costa et al., 2002; Sharma, 2005; Rodenhiser and Mann, 2006), and Rett syndrome, mostly caused by a mutation in the gene MeCP2 that produces methyl-CpG- binding protein, is an X linked dominant neurodevelopmental disorder (Bienvenu et al., 2000). The loss of the MeCP2 protein results in loss of transcriptional repression and hence inappropriate transcription of downstream genes during brain development (Van Den Veyver and Zoghbi, 2001; Rodenhiser and Mann, 2006).

### Infectious diseases

One of the most promising strategies to treat an infectious disease is to replace a receptor on a host target cell, which is used by a pathogen to gain entry into the cell, with a mutated one. This makes the receptors on the target cell non-functional and blocks the entry of the pathogen into the cell, preventing its multiplication and the progression of the disease. One important disease that may eventually be universally treated and perhaps eradicated this way is acquired immunodeficiency syndrome (AIDS). AIDS is a disease of the human immune system caused by infection with human immunodeficiency virus (HIV). It is a major public health concern worldwide, with more than 30 million people infected (Kallings, 2008; Unaids, 2013). There is no cure or vaccine at present, but antiretroviral treatment (ART) can slow the course of the disease and result in near-normal life expectancy. ART reduces the risk of death and complications from the disease, but is expensive and associated with side-effects. We need to find better treatments.

Of note, one of the molecular mechanisms of HIV infection and resulting AIDS is depletion of CD4^+^ T cells. All cells susceptible to HIV infection (CD4 T cells, macrophages, and dendritic cells) are derived from hematopoietic stem cells. Following infection, most CD4^+^ T cell loss occurs in the intestinal mucosa which harbors the majority of the lymphocytes found in the body (Mehandru et al., 2004).

C-C chemokine receptor type 5, also known as CCR5, is a protein on the surface of white blood cells. It functions as a receptor for chemokines allowing T cells to hone in on target tissues and organs. HIV often uses CCR5 to enter and infect host cells. The majority of mucosal CD4^+^ T cells express the CCR5 protein, which HIV uses as a co-receptor to gain access to the cells (Brenchley et al., 2004). HIV seeks out and destroys CCR5-expressing CD4^+^ T cells during acute infection. However, certain individuals carry a mutation known as CCR5-Δ32, which renders the receptor non-functional and prevents entry of HIV into the cells. In humans the CCR5 gene is located on chromosome 3. A Δ32 mutation results in the genetic deletion of a portion of the CCR5 gene. Homozygous carriers of this mutation are resistant to certain strains of HIV-1 infection (Samson et al., 1996). This mutation, although having negative effects on T-cell function, can protect these individuals against HIV.

Future reprogramming technology may allow generation of CCR5-deficient HSCs derived from hESCs/iPSCs, perhaps by creating iPSCs from a CCR5-deficient individual (Ledran et al., 2008; Hutter et al., 2009), or even better from an existing patient using gene editing technology. Diseases such as HIV could potentially be cured by means of hematopoietic cell transplantation whereby HSCs or even fibroblasts are first dedifferentiated into continuously growing iPSC lines using induced reprogramming, followed by gene editing to introduce the Δ32 mutation and then directed differentiation toward the desired hematopoietic lineage followed by bone marrow transplantation (Kambal et al., 2011). The use of engineered zinc finger nucleases (ZFNs) to disrupt the CCR5 gene in human HSCs *in vivo* suggested that ZFN mediated autologous HSC modification could provide a permanent supply of HIV-resistant CD34^+^ HSC progeny which as well would be capable of immune reconstitution and long-term protection against viral replication (Holt et al., 2010).

In principle this basic idea could be applied to other infectious diseases that rely on a single or even multiple receptors to gain entry into human cells.

## Conclusion

The last decade has seen a boom in the field of stem cell biology and regenerative medicine coupled with extensive studies on the epigenetics involved in cellular reprogramming and disease. Nuclear reprogramming experiments beginning in the early 1950's introduced the concept of generation of pluripotent cells from somatic cells through nuclear transfer. The discovery of hESCs in the late 1990's acquainted us with a source for designing therapies to treat a broad range of diseases. Following that came the invention of iPSC technology, bringing stem cell production and its use in medicine out of an ethical quandary. Today, stem cell therapies have advanced into clinical trials and we predict that it will not be long before regenerative medicine units become established at health care centers.

Similarly, epigenetic studies have revolutionized the classical genetic approach toward studying and treating hereditary diseases. As we embark on this journey to understand every aspect of the interlocking pathways and processes underlying aging, cancer, regeneration, and repair, we still need to completely decipher the epigenetic code in order to design and implement effective and safe stem-cell based therapies to treat age-related diseases, acute injuries and perhaps chronic disorders.

A full understanding of epigenetics, reprogramming, senescence, cancer, and regeneration will drive remarkable progress in achieving cellular and rejuvenation therapies as well as designing drugs for diseases with an epigenetic basis. The best approach is to embrace all of these fields in a unifying synthesis.

### Conflict of interest statement

The authors declare that the research was conducted in the absence of any commercial or financial relationships that could be construed as a potential conflict of interest.
